# Supplementary material for: Genome-Wide Characterization and Expression Profiling of the AP2/ERF Gene Family in Fragaria vesca L
Source: Int J Mol Sci. 2024 Jul 11;25(14):7614. doi: 10.3390/ijms25147614 (PMC11277216; doi:10.3390/ijms25147614)
Supplement: Supplementary file 1 [file ijms-25-07614-s001.zip › Supplementary Figures.pdf]

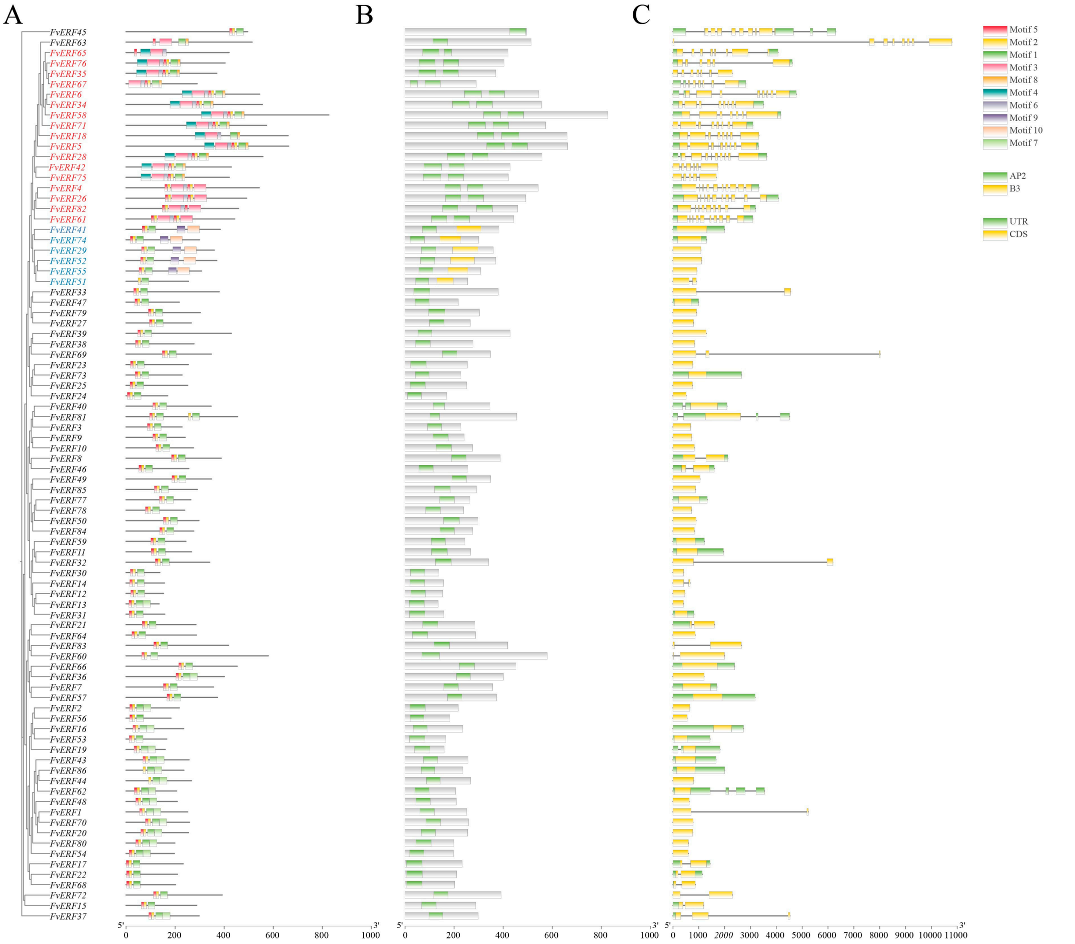

Figure S1. *Fragaria vesca* AP2/ERF superfamily evolutionary tree with conserved motifs, domain and gene structure analysis. (**A**) Constructing the unrooted neighbor-joining (NJ) tree using MEGA11 as a foundation, with bootstrap values tested 1000 times. AP2 subfamily members are denoted in red font, RAV subfamily members in blue font, and the remaining ERF subfamily and Soloist members in black font. The size and position of different color blocks correspond to the position of motif 1-10 on the protein, respectively. (**B**) The gray areas represent the non-conserved structural domains of the *FvAP2/ERF* genes, whereas the colored areas correspond to the conserved structural domains. (**C**) Distribution of intron and exon structures of the *FvAP2/ERFs*. The green part represents the exonic region, the yellow part the untranslated region, and the gray line the intronic part.

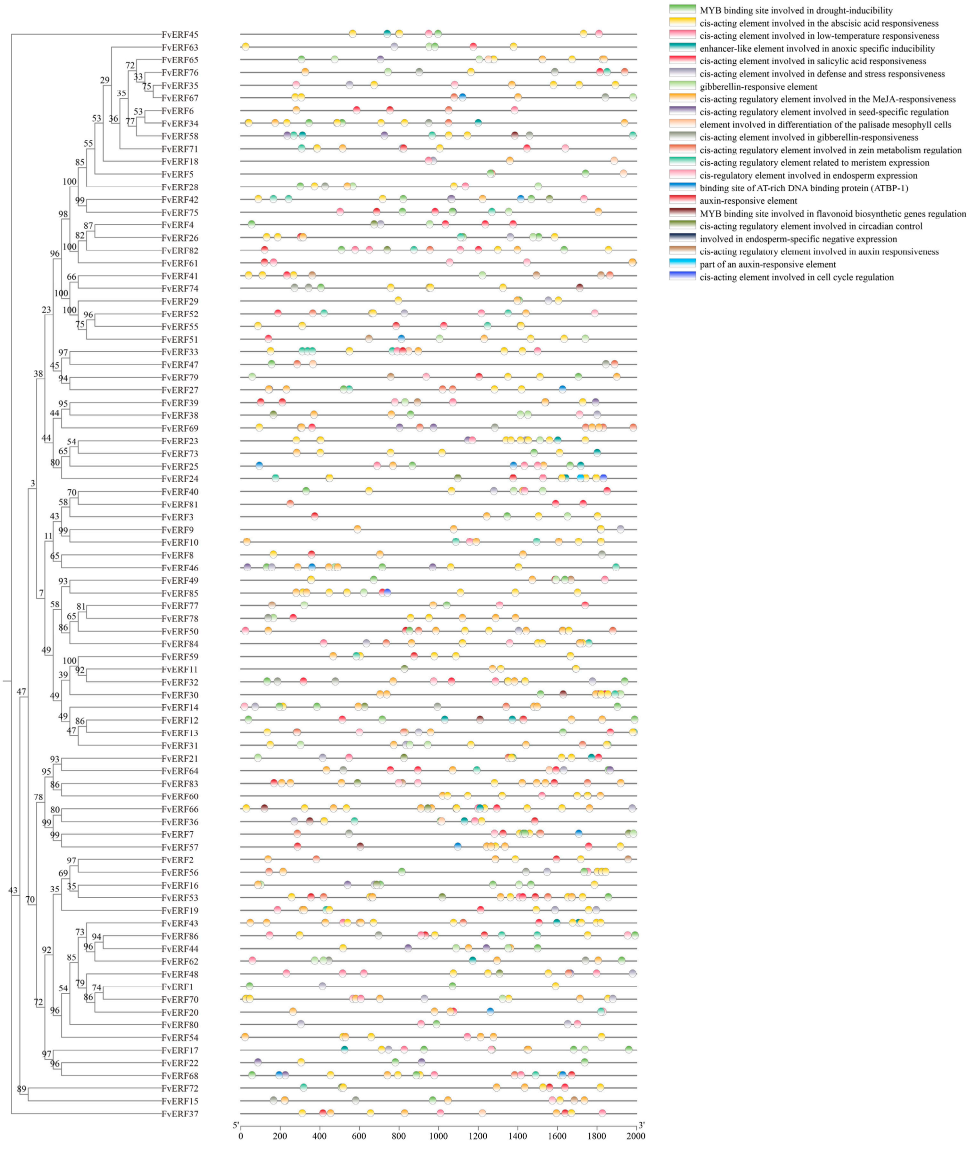

Figure S2. In *Fragaria vesca*, the AP2/ERF genes' cis-acting elements (screened).

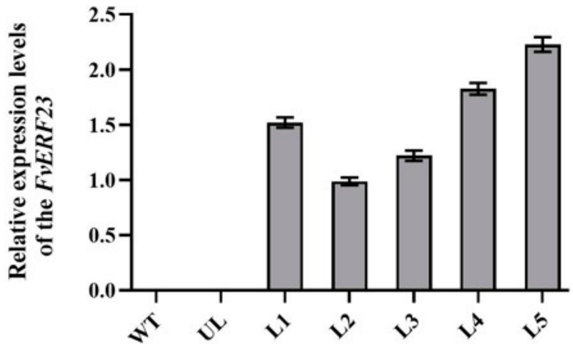

Figure S3. RT-qPCR analysis was conducted to determine the relative expression levels in wild-type (WT) *Arabidopsis*, unloaded (UL) *Arabidopsis*, and transgenic *Arabidopsis* overexpressing *FvERF23*.
